# Clinically feasible *B*_1_ field correction for multi‐organ sodium imaging at 3 T

**DOI:** 10.1002/nbm.4835

**Published:** 2022-10-11

**Authors:** Michael Vaeggemose, Rolf F. Schulte, Christoffer Laustsen

**Affiliations:** ^1^ GE Healthcare Brondby Denmark; ^2^ MR Research Centre, Department of Clinical Medicine Aarhus University Aarhus Denmark; ^3^ GE Healthcare Munich Germany

**Keywords:** *B*_1_ field correction, clinically feasible protocol, multi‐organ, sodium imaging

## Abstract

Sodium MRI allows the non‐invasive quantification of intra‐organ sodium concentration. RF inhomogeneity introduces uncertainty in this estimated concentration. *B*_1_ field corrections can be used to overcome some of these limitations. However, the low signal‐to‐noise ratio in sodium MRI makes accurate *B*_1_ mapping in reasonable scan times challenging. The study aims to evaluate Bloch–Siegert off‐resonance (BLOSI) *B*_1_ field correction for sodium MRI using a 3D Fermat looped, orthogonally encoded trajectories (FLORET) read‐out trajectory.

We propose a clinically feasible *B*_1_ field map correction method for sodium imaging at 3 T, evaluating five healthy subjects' brain, heart blood, kidneys, and thigh muscle. We scanned the subjects twice for repeatability measures and used sodium phantoms to determine organ total sodium concentration. Conventional proton scans were compared with sodium images for organ structural integrity. The BLOSI approach based on the 3D FLORET read‐out trajectory was used in *B*_1_ field correction and 3D density‐adapted radial acquisition for sodium imaging.

Results indicate improvements in sodium imaging based on *B*_1_ field correction in a clinically feasible protocol. Improvements are determined in all organs by enhanced anatomical representation, organ homogeneity, and an increase in the total sodium concentration after applying a *B*_1_ field correction.

The proposed BLOSI‐based *B*_1_ field correction using a 3D FLORET read‐out trajectory is clinically feasible for sodium imaging, which is shown in the brain, heart, kidney, and thigh muscle. This supports using fast *B*_1_ field mapping in the clinical setting.

## Graphical abstract

We propose a clinically feasible *B*_1_ field map correction method for sodium imaging at 3 T. The method is evaluated in the brain, heart blood, kidneys, and thigh muscle of five healthy subjects. The subjects were scanned twice for repeatability measures. The Bloch–Siegert off‐resonance approach based on a 3D Fermat looped, orthogonally encoded trajectories readout trajectory was used for *B*_1_ field correction and 3D density‐adapted radial acquisition for sodium imaging.

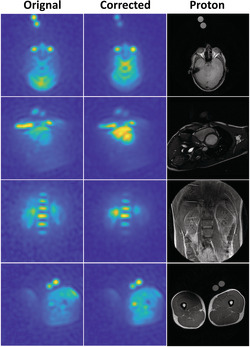

**Abbreviations**
^1^H: proton
^13^C: carbon
^23^Na: sodium
^31^P: phosphorus
*B*_1_^−^
: receive magnetic field
*B*_1_^+^
: transmit magnetic field
BLOSI: Bloch–Siegert off resonance
CS: compressed sensing
FLORET: Fermat looped, orthogonally encoded trajectories
PROPELLER: periodically rotated overlapping parallel lines with enhanced reconstruction
ROI: region of interest
SNR: signal‐to‐noise ratio
TSC: total sodium concentration

## 1 INTRODUCTION

Sodium (^23^Na) MRI allows the non‐invasive quantification of intra‐organ sodium concentration in vivo,^1^ a concentration that is coupled with electrophysiological and metabolic processes crucial to life.^2^, ^3^ The energy‐dependent ion gradient controlled by the sodium‐potassium pump has been suggested as a biomarker of cellular metabolic energy deficits.^4^ The MR signal observed combines the intracellular and extracellular compartments, and often represents changes in their relative distribution.^5^, ^6^, ^7^ The relative increase in ^23^Na signal is attributed to several factors, including cell death.^4^ Although ^23^Na imaging is a well known technique, it has not transitioned to widespread clinical use.^8^, ^9^ This is likely because the inherently low signal‐to‐noise ratio (SNR) of ^23^Na MRI is an intrinsic limiting factor of the technique,^5^, ^8^ with, for example, cardiac ^23^Na imaging possessing an SNR 6000 times lower than that of protons (^1^H).^10^ Technical advances such as advanced, short *T*_E_ imaging readouts and the increased use of 7 T MRI scanners have given ^23^Na imaging a renewed interest.^5^

A challenge for quantitative ^23^Na imaging is the varying transmit and receive *B*_1_ fields of the used RF coil, which are commonly ^23^Na surface coils.^11^, ^12^, ^13^ Homogeneous transmit (*B*_1_^+^) and receive (*B*_1_^−^) magnetic fields are necessary for the production of high‐quality MR images and reliable signal quantification. RF coils used in transmit mode create a magnetic field, *B*_1_^+^, to rotate the net magnetization according to the desired flip angle for signal acquisition. Receive coils acquire signal from changes in the magnetic flux, which is subsequently reconstructed as images or spectra. The *B*_1_^−^ field describes the coil receive sensitivity profile, which, like *B*_1_^+^, depends on the geometry of the coil element used. As these fields are typically strong functions of space, moving the object of interest away from a single coil element reduces *B*_1_^±^ and hence the coil's sensitivity. Similarly, any *B*_1_^+^ or *B*_1_^−^ field inhomogeneity arising due to either the geometry of the coil elements or the electrodynamic interactions of the sample in the scanner will result in acquired and unwanted signal variation across the excited field of view, which is often quantified in microtesla. Compensating for these imperfections requires knowledge of the sensitivity profiles or added scan time. Fortunately, coil transmit and receive sensitivity profiles, according to principle of reciprocity,^14^, ^15^ are approximately the same at 33 MHz, the resonance frequency of ^23^Na at 3 T. *B*_1_ field corrections can be applied to compensate for these variations. Correction methods aim to measure *B*_1_, and typically rely on the relation between pulse length, RF field amplitude,^16^, ^17^ flip angle,^18^ acquired signal magnitude,^19^, ^20^ and signal phase.^21^, ^22^

Multi‐nuclear imaging experiences a lower signal sensitivity than ^1^H imaging.^23^ In order to increase SNR, low resolution data obtained with an increased number of averages is often acquired. These approaches to improve SNR are problematic for widespread clinical use, especially if information is required rapidly for treatment decisions.^24^ As a consequence, regularized *k*‐space undersampling has been used in an attempt to both reduce scan time and preserve SNR, without deleteriously affecting the quantification of the sodium concentration. Approaches include Tikhonov regularization,^25^ trained convolutional neural networks,^26^ or compressed sensing (CS),^27^ where some CS methods require additional information about coil sensitivity. An additional challenge is that ^23^Na sequences with a short repetition time and a high number of acquisitions deposit a large amount of RF energy. These factors together make the application of clinically feasible *B*_1_ field corrections challenging, although still proven possible in ^23^Na,^11^, ^12^, ^28^ carbon (^13^C),^29^, ^30^, ^31^ and phosphorus (^31^P)^32^, ^33^ imaging. Owing to its short *T*_2_*, ^23^Na imaging requires a short echo time, further limiting the number of *B*_1_ mapping techniques. The double angle,^11^ phase‐sensitive^12^ and Bloch–Siegert shift^22^ methods are feasible possibilities, where the double angle method is the most common. The Bloch–Siegert off‐resonance pulse approach (BLOSI)^22^, ^34^ is reported to be faster and independent of *T*_1_ effects, and yields higher SNR than the double angle approach.^11^, ^12^ The aim of this study is to evaluate BLOSI *B*_1_ field correction of ^23^Na images in healthy human thigh muscle, heart, kidney, and brain using a 3D Fermat looped, orthogonally encoded trajectories (FLORET) read‐out trajectory.^35^

## 2 MATERIALS AND METHODS

### 2.1 Study population

Through public announcements, we recruited five healthy subjects. Healthy is defined as having no medical history in the organs of interest (brain, heart, kidneys, and thigh muscle) and no use of medicine besides contraceptives and vitamins. All study participants gave informed consent and were examined between June 2021 and November 2021. Subjects included were aged 34 (26–42) years, weight 70 (45–94) kg, height 176 (163–190) cm, with a gender distribution of 60% male. The local ethics committee (no 1‐10‐72‐210‐21) and ClinicalTrials.gov (no NCT05215938) approved the study.

### 2.2 BLOSI

BLOSI measures a phase difference induced by an off‐resonance pulse applied after excitation.^36^ This phase difference stems from the Bloch–Siegert effect, which is related to the applied transmit *B*_1_ field^22^ and described by the following equations:

(1) $$\varphi_{\mathrm{BS}}=B_{1,\text{peak}}^{2}\int_{0}^{T}\frac{{\left(\gamma B_{1,\text{normalized}}\left(t\right)\right)}^{2}}{2\omega_{\mathrm{RF}}\left(t\right)}dt=B_{1,\text{peak}}^{2}K_{\mathrm{BS}}$$

(2) $$B_{1}\left(t\right)=B_{1,\text{peak}}B_{1,\text{normalized}}\left(t\right).$$

Here, $K_{\mathrm{BS}}$ is the Bloch–Siegert phase shift constant that is evolved by a time‐dependent and normalized RF pulse shaped transmit field ( $B_{1,\text{normalized}}\left(t\right)$) of total pulse duration T. ^22^

After the RF excitation pulse, the Bloch–Siegert pulse induces a *B*_1_^+^ dependent phase shift. Applying at least two acquisitions with plus and minus the BLOSI off‐resonance frequency undesired phase effects arising from other sources can be removed, and thus *B*_1_^+^ measured.^22^

The BLOSI pulse used in conjunction with the FLORET sequence is a slice‐selective pulse‐acquire sequence using a Fermi‐shaped off‐resonance pulse, which starts directly after the excitation pulse, followed by the slice refocusing gradient.^36^ BLOSI parameters include the off‐resonance pulse (±2000 Hz) applied for a *T* = 4 ms duration. The BLOSI pulse amplitude is scaled (~0.3436) by a factor according to the initial excitation pulse to ensure correct RF transmit excitation. The phase shift constant for the Fermi pulse is $K_{\mathrm{BS}}$ = 0.0287522 rad/G^2^. The number of off‐resonance pulses in the FLORET is 198, with 396 as the total number of scans. According to the reciprocity principle coil transmit and receive sensitivity profiles are approximately the same for ^23^Na at 3 T (33 MHz).^14^, ^15^ In the results, *B*_1_ field corrections combine the transmit (*B*_1_^+^) and receive (*B*_1_^−^) fields.

### 2.3 MRI

MRI was acquired on a 3 T MRI scanner (GE MR750, GE Healthcare, Waukesha, WI, USA), allowing ^1^H MRI as well as ^23^Na imaging. A commercial Helmholtz coil pair (PulseTeq, Guildford, UK) was used for ^23^Na imaging. The diameter of the coil loops was 20 cm. ^23^Na in the thigh muscle, heart, kidneys, and brain was imaged. Five healthy subjects participated. Repeatability was evaluated by scanning each subject twice at least two weeks apart. ^1^H images were acquired with a 2D periodically rotated overlapping parallel lines with enhanced reconstruction (PROPELLER)^37^ for anatomical reference and a *B*_0_ field map to evaluate the static field quality. RF pulses (transmit gain and centre frequency) were calibrated to optimize the ^23^Na imaging acquisition. A *B*_1_ transmit field map was acquired using a BLOSI approach^22^ based on the FLORET technique.^35^ ^23^Na images were acquired with a density‐adapted radial acquisition technique, as described by Nagel et al,^38^ and zero‐filled by a factor of 2 in all three dimensions. The read‐out bandwidths were 125 kHz and 33.3 kHz in the FLORET and density‐adapted ^23^Na sequences, respectively. The imaging protocol's scan parameters are listed in Table 1 and applied for both phantoms and in vivo examinations.

**TABLE 1:** Scan protocol of ^1^H and ^23^Na imaging sequences

Sequence	*T*_R_ (ms)	FA (°)	*T*_E_ (ms)	FOV (cm)	Matrix	*N*_EX_	Scan time (min:s)
Localizer	—	—	1.4	48	256 × 128	—	00:26
*B*_0_‐map IDEAL	5.5	3	4.98	35	128 × 128	1	00:18
2D PROPELLER	2800	111	25.4	35	256 × 256	1	01:33
BLOSI	250	90	4.75	35	—	1	00:08
*B*_1_‐map FLORET	160	45	4.75	35	30 × 30	2	02:07
3D radial	5.8	20	0.31	35	35 × 35	32	11:59

*T*_R_, repetition time, FA, flip angle, *T*_E_, echo time, FOV, field of view, *N*_EX_, number of excitations.

### 2.4 Image processing and analysis

**FIGURE 1:**
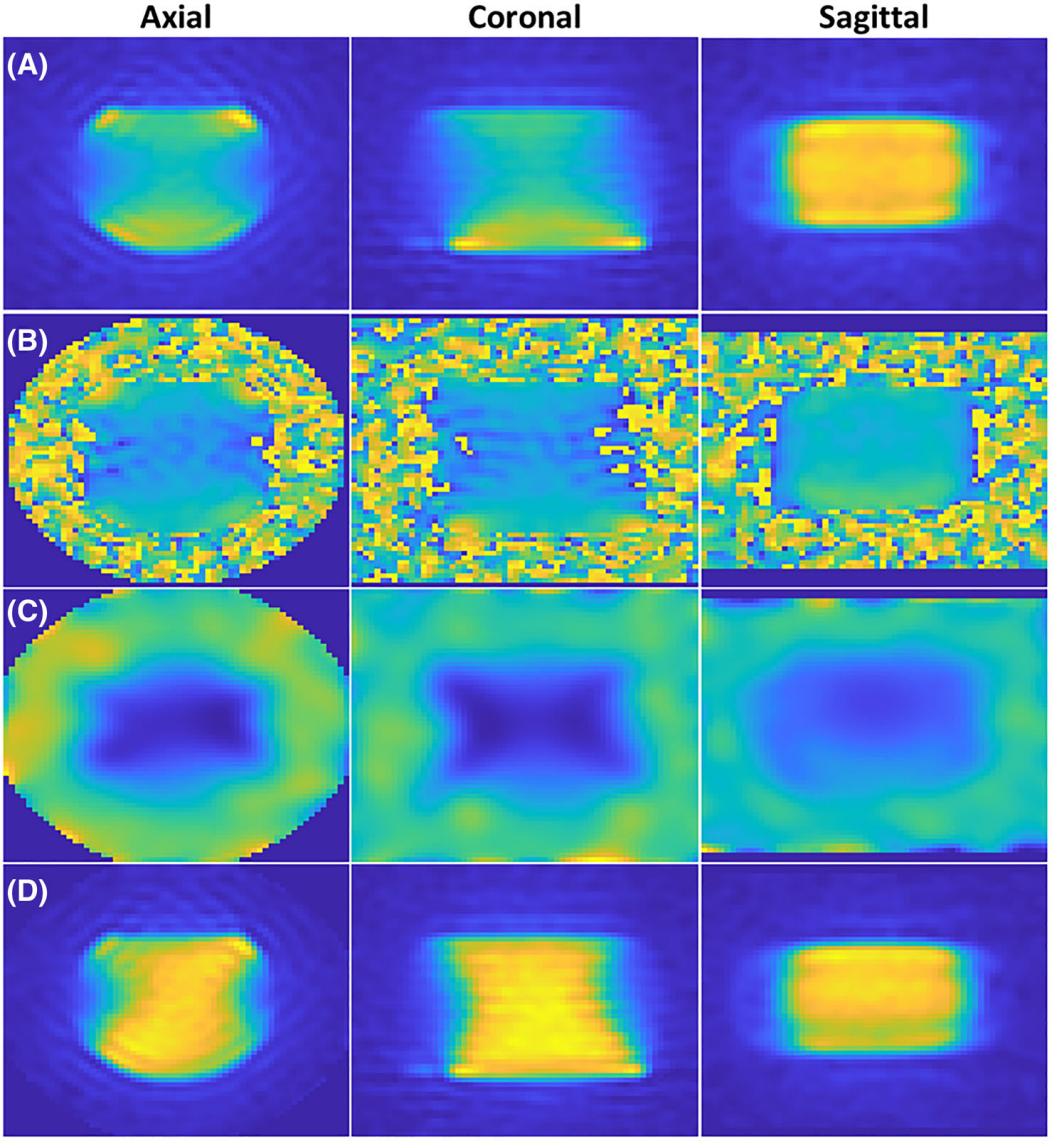
Overview of the *B*_1_ field correction image processing pipeline on saline water phantoms. A, The original images in the axial, coronal, and sagittal orientations. B, *B*_1_ field map as calculated from the FLORET Bloch–Siegert method. C, We apply a voxel‐wise polynomial fitting and extrapolation to reduce noise. D, The *B*_1_ field corrected images in the corresponding orientations.

The method was evaluated in a saline water phantom. The phantom holds 40 g NaCl dissolved into a bottle containing 10 L of tap water, resulting in a 27 mmol/L sodium concentration. Reference phantoms consisted of 0.1 g in 50 ml (14 mmol/L) and 0.4 g in 50 ml (54 mmol/L). For in vivo measurements, sodium phantoms were placed in the field of view (32 and 80 mmol/L, 4% agar) to determine total sodium concentration (TSC). The phantoms were placed in the middle of the anterior coil element of the two loops. Sodium concentrations were determined by mapping the voxel‐by‐voxel values of the image to a linear regression fit between the two sodium phantoms and noise.^39^ The slope and offset were derived from a linear regression model and used for sodium concentration calculations by applying the following linear correction: sodium concentration = (signal − offset)/slope [mmol/L]. A voxel‐wise polynomial fitting and extrapolation was applied to the calculated *B*_1_ maps to reduce noise. Images were processed and analysed in MATLAB R2020a (MathWorks, Natick, MA, USA) using the multi‐nucleus‐spectroscopy research pack created by GE Healthcare. An overview of the image processing pipeline is shown on the saline water phantom in Figure 1.

### 2.5 Statistical analysis

*B*_1_ field correction effects were evaluated based on anatomical structure integrity, repeatability, and signal homogeneity inside selected organs. Repeatability was estimated from SNRs of the high concentration phantom at the initial and follow‐up scan. Accurate anatomical structure was determined from ^1^H images and homogeneity from standard deviations of histogram probabilities. Multiple paired *t*‐tests were performed on repeatability measures and the standard deviations of histogram probabilities acquired from the original and corrected ^23^Na images. The level of significance was adjusted for multiple comparisons with the Bonferroni–Dunn method. Organ specific sodium concentrations were compared using the two‐way ANOVA for multiple comparisons and *p*‐values were Bonferroni corrected. GraphPad Prism 8.0.0 (GraphPad Software, San Diego, CA, USA) was used for statistical analyses.

## 3 RESULTS

**FIGURE 2:**
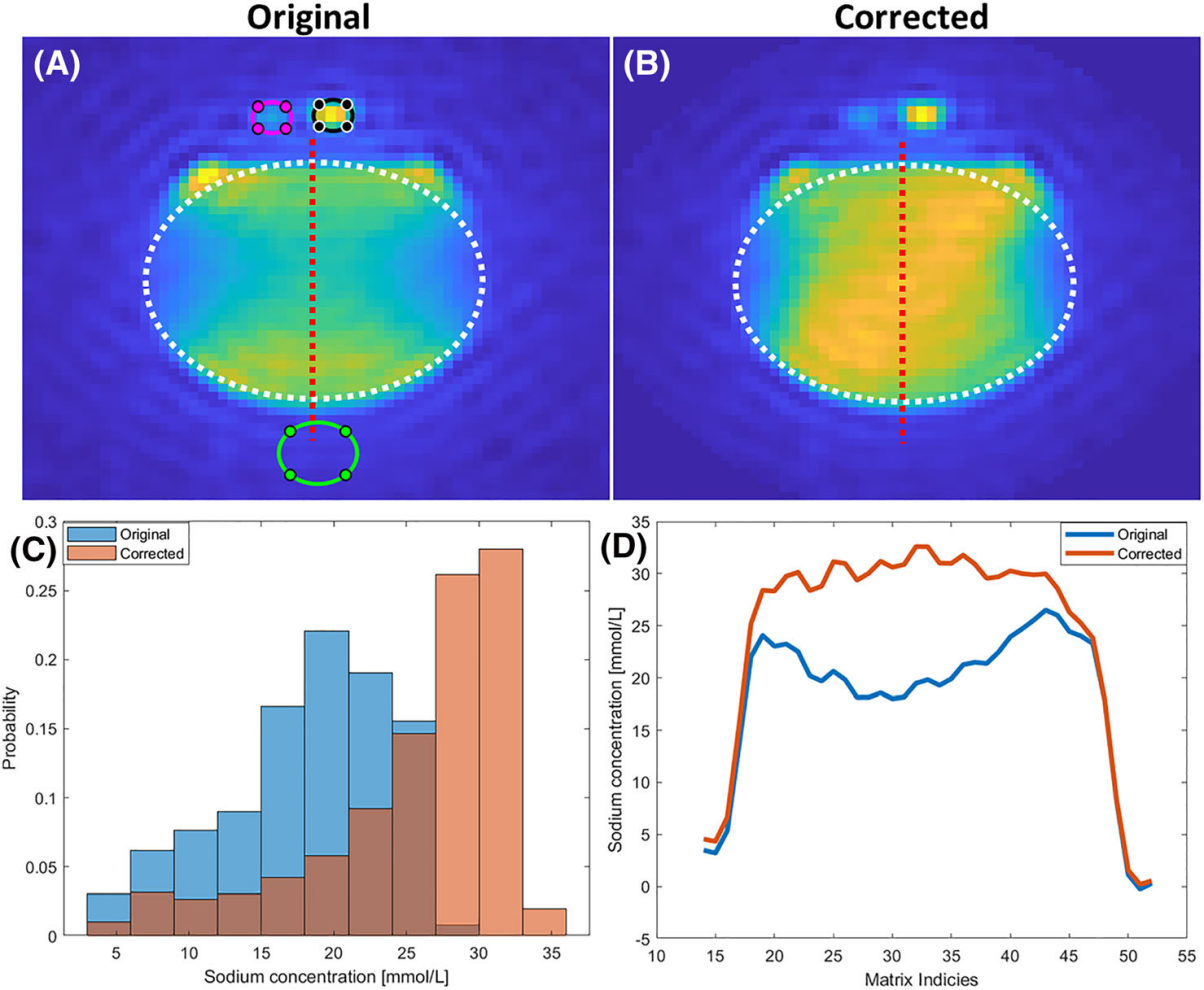
Saline water phantom for evaluation of the method. A, B, Original (A) and *B*_1_ field corrected (B) ^23^Na images of the brain. The white ellipses indicate an ROI. C, The histogram's sodium concentration probabilities indicate increased uniformity and an increased number of voxels where the sodium concentration is closer to the correct value (27 mM). D, The sodium concentration across the phantom (red dotted line in A and B) of the original and corrected image. Reference phantoms: low concentration (magenta), high concentration (black), and noise (green) ROIs (see A).

The original and corrected images of the saline water bottle with a white, elliptical region of interest (ROI) and a red dotted line are shown in Figure 2A and 2B. Low (magenta) and high (black) concentration phantoms are illustrated in addition (Figure 2A). The histogram of the ellipse (Figure 2C) points to an increase in sodium levels towards the correct concentration of 27 mmol/L. From the red dotted line, a cross‐sectional plot highlights the rise in sodium levels towards a more homogeneous signal across the phantom.

**FIGURE 3:**
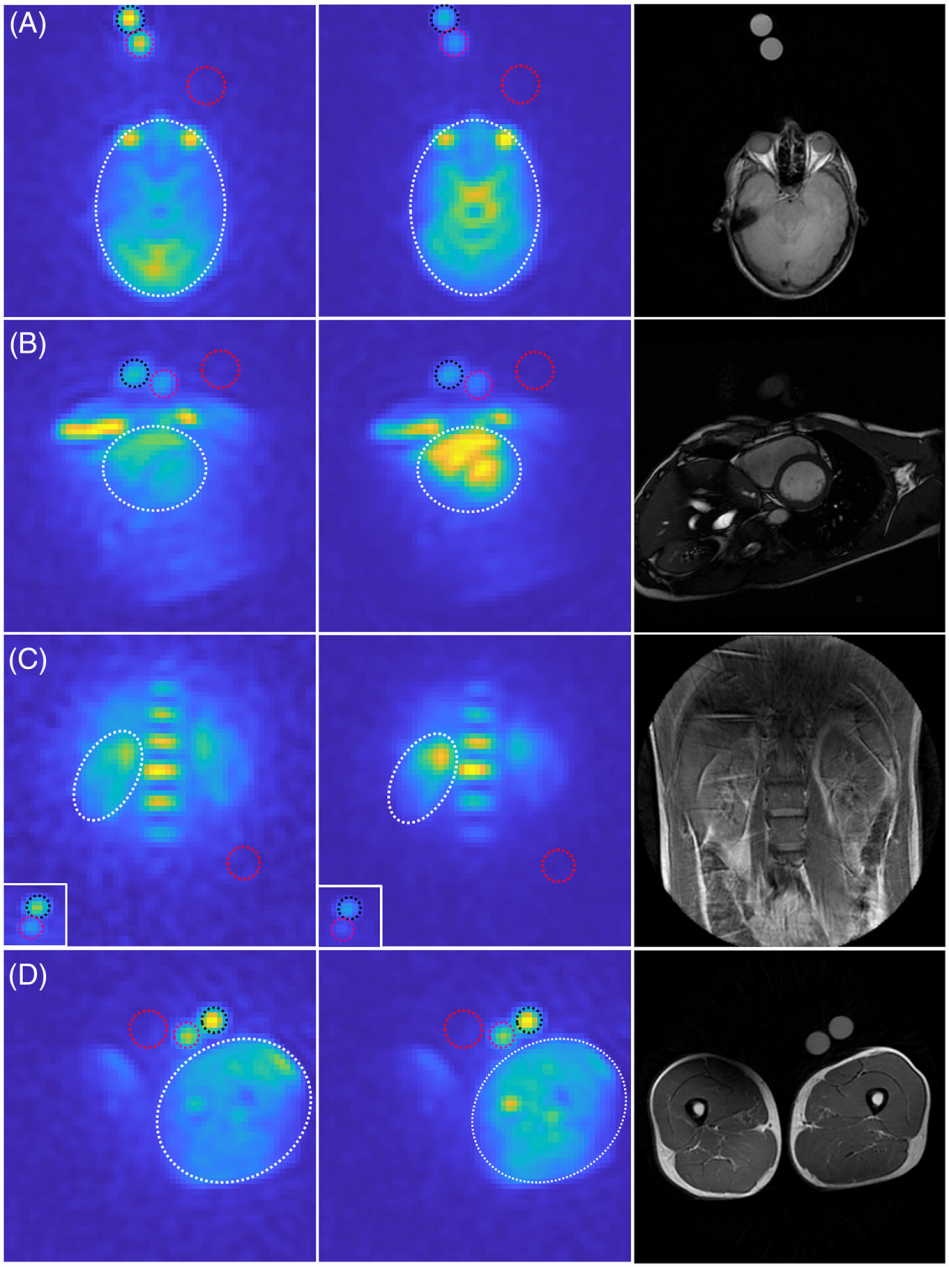
*B*_1_ field correction of ^23^Na images: original (first column), corrected (middle column), and ^1^H image (last column). Anatomies: A, brain; B, heart; C, kidney; D, thigh muscle. Circles are the organ ROI (white), 32 mmol/L phantom (magenta), 80 mmol/L phantom (black), and noise (red).

Visual inspection of the in vivo *B*_1_ field corrected images indicated an increased ^23^Na signal in the organ of interest, which was most pronounced in the brain (Figure 3A). Structural improvements were apparent in all anatomies compared with ^1^H images (Figure 3, last column). ^23^Na images showed a high natural abundance in the blood indicated by the two cardiac chambers (Figure 3B), the bone marrow in the ribs (Figure 3B), the vertebral column (Figure 3C), and large vessels in the thigh muscle (Figure 3D).

**FIGURE 4:**
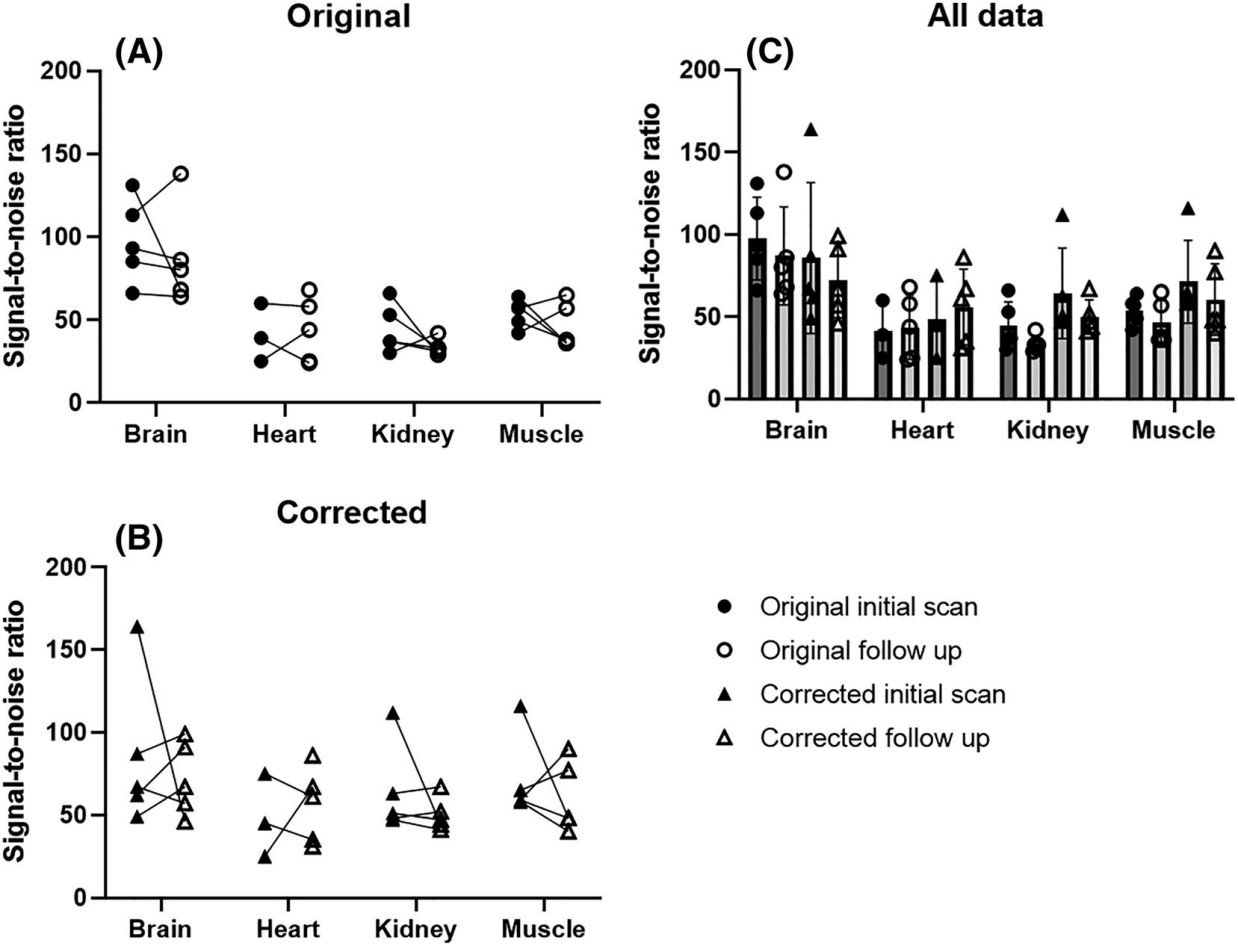
C, Sodium phantom SNR in original and corrected data at initial and follow‐up scan at different anatomical locations (thigh muscle, kidney, heart, and brain). A, B, Individual subject variation before (A) and after (B) *B*_1_ field correction is shown in separate plots.

Sodium phantom SNR was measured in each data set and compared between initial and follow‐up scans to determine repeatability (Figure 4). Results show minor changes in SNR as compared with the original images on a group level (Figure 4C). A similar minor variation is measured on a subject level in both original and corrected images (Figure 4A and 4B). No significant differences between initial and follow‐up scans were determined using multiple paired *t*‐tests on variation after Bonferroni–Dunn correction.

**FIGURE 5:**
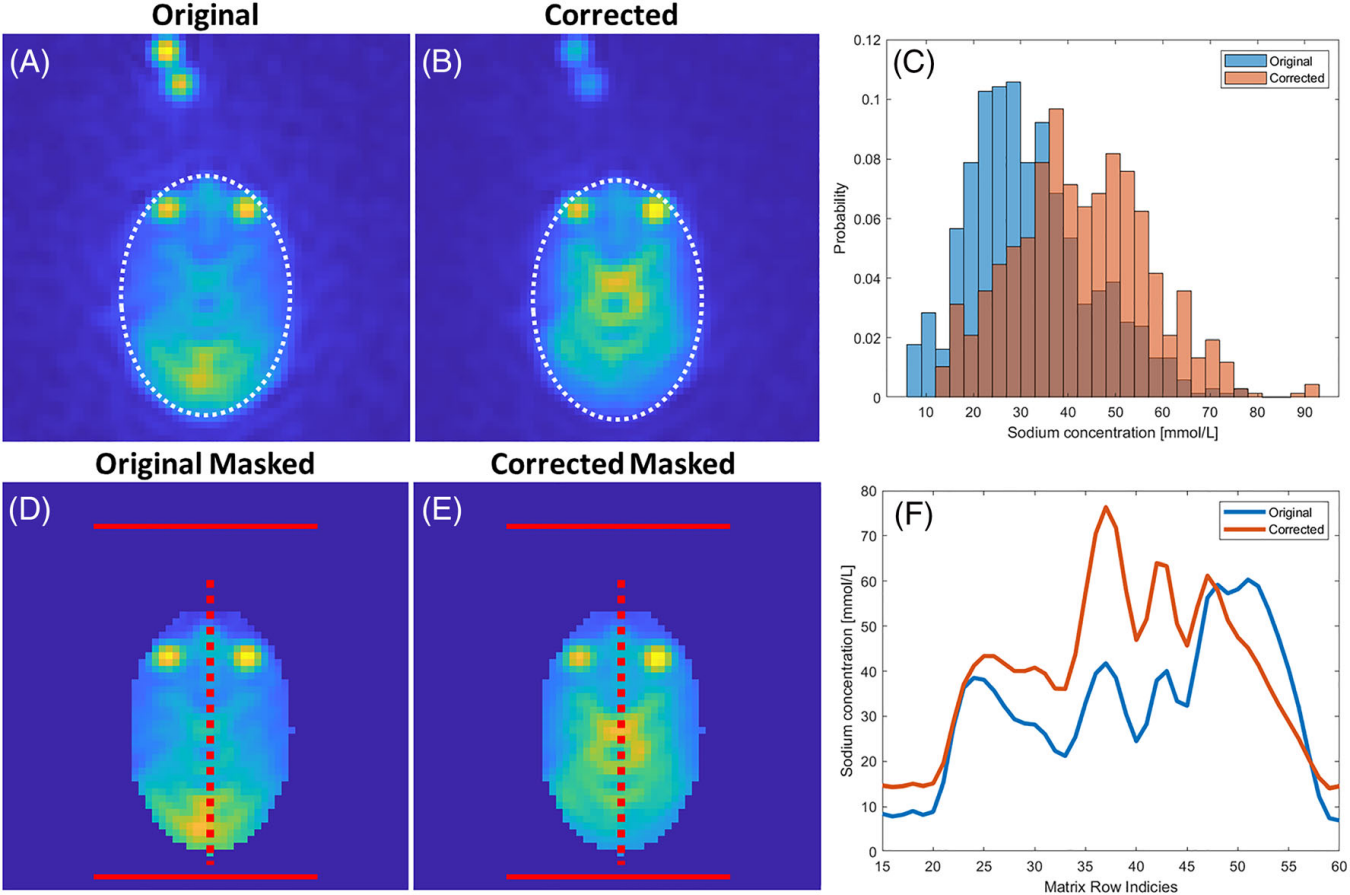
Comparison of the brain's original (first column) and *B*_1_ field corrected (second column) ^23^Na images. A, B, The white ellipses indicate ROI in the original image (A) and the corrected image (B). C, ROI histograms are shown as sodium concentration probabilities to indicate ROI homogeneity. D, E, The original and corrected images masked according to the ROI. F, Plot of the sodium concentration across the red dotted line from the original and corrected images.

Image homogeneity from *B*_1_ corrections is quantified with an ROI at each anatomy (Figure 3, white ellipses). Results from the brain analysis are shown as the TSC variation per histogram (Figure 5C) and in a cross‐sectional line (Figure 5F).

The TSC values in the central part of the brain indicate a variation in the naturally occurring sodium concentrations. TSC levels are highest in the eyes as compared with adjacent tissue types (Figure 5). TSC homogeneity was determined from the standard deviations of histogram probabilities (Figure 5C).

**FIGURE 6:**
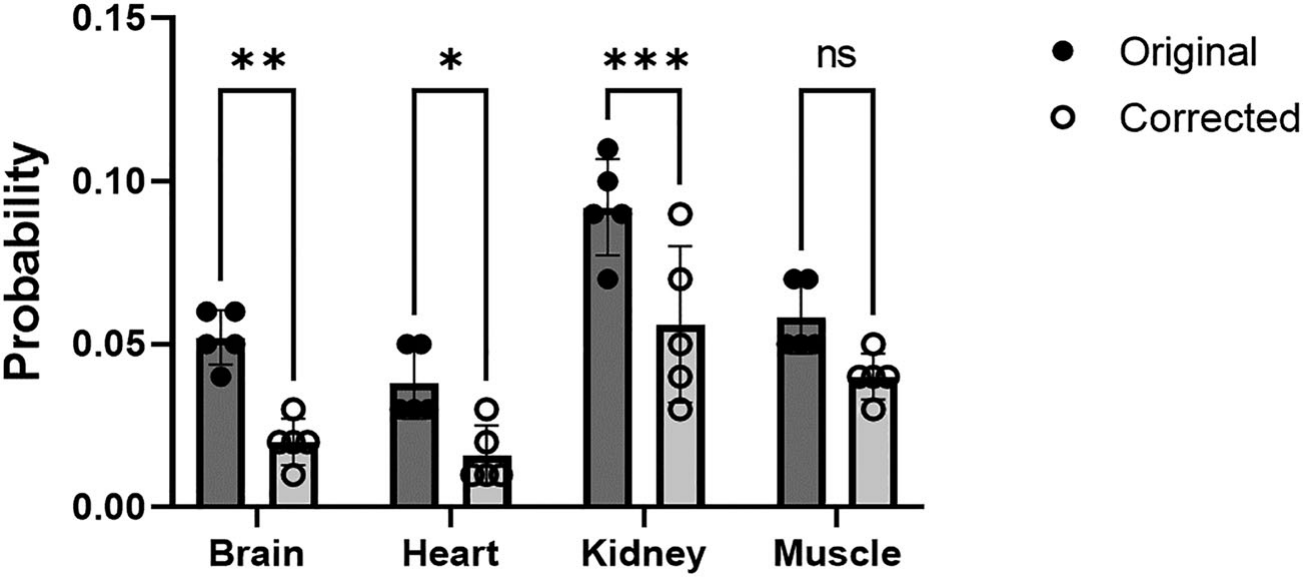
Standard deviation of histogram probabilities before and after *B*_1_ field correction at different anatomical locations (brain, heart blood, kidney, and thigh muscle). Asterisks indicate the level of significance after Bonferroni–Dunn correction: ***p < 0.001, **p < 0.01, *p < 0.05, ns, not significant.

The results indicate a lower variation in the histogram probabilities of all organs. After the Bonferroni–Dunn correction, this was significant in the brain, heart blood, and kidneys (Figure 6).

**FIGURE 7:**
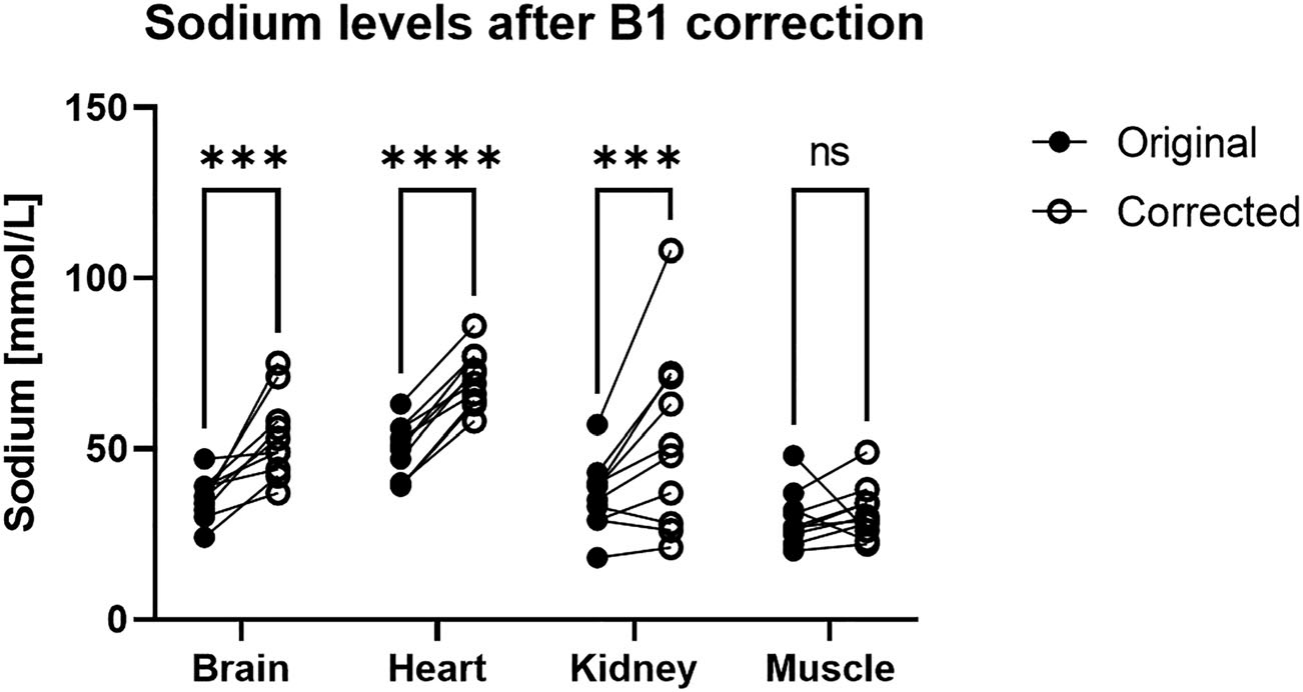
Comparison of sodium level concentrations before and after *B*_1_ field correction. Asterisks indicate the level of significance after Bonferroni correction. ****p < 0.0001, ***p < 0.001, ns, not significant.

Analysing TSC after *B*_1_ field correction indicates a significant increase in the brain, heart blood, and kidneys after Bonferroni correction (Figure 7).

## 4 DISCUSSION

The study shows the clinical feasibility of a simple and fast *B*_1_ field correction method to correct ^23^Na images of the brain, heart blood, kidneys, and thigh muscle. The implemented BLOSI approach using a 3D FLORET read‐out trajectory eliminates *T*_1_‐induced effects, in contrast to commonly used double angle acquisitions.^11^, ^12^ Results indicate improved anatomical representation and organ homogeneity and increased TSC after applying *B*_1_ field correction.

The method was validated in a saline water bottle phantom, showing improved TSC quantification and signal homogeneity. Assessing organ anatomy by comparing ^1^H and ^23^Na images displayed structural improvements in the ^23^Na images, suggesting improvement from *B*_1_ field correction. The gain from the modification was increased according to the distance of coil elements from the ROI. Brain ^23^Na images are an excellent example of increasing the signal in the front and reducing it in the back (Figure 5).

Participating subjects were healthy with an expected constant normal distribution of TSC within the examined tissue types. From this assumption, we evaluated *B*_1_ field correction effects from the width (standard deviation) of derived TSC histograms. We considered the quantification of tissue TSC to be improved if this standard deviation decreased. Our results show distributions with less variation, which indicates enhanced *B*_1_ field homogeneity in the corrected images of brain, heart blood, and kidneys. The standard deviation of TSC was lower in the *B*_1_ corrected thigh muscle images; however, no significant difference was determined. The normal distribution assumption is limited in the kidney and the brain as it consists of tissue types with different TSCs. Nevertheless, indications of tissue separation are seen in the histogram (Figure 5C) with peaks at 35 mmol/L and 50 mmol/L, corresponding to mean values for white and grey matter TSC, respectively.^8^

Reproducibility was determined from the change in SNR of sodium phantoms between the initial and follow‐up scans. SNR results from original and *B*_1_ field corrected images showed no significant differences, indicating good reproducibility of the ^23^Na imaging methods.

The quantitative TSC values show an increase in the brain, heart blood, and kidneys when applying *B*_1_ field correction. Comparing the corrected TSC values with the literature, we find a good agreement.^8^, ^9^ This is seen in the brain, 53 ± 12 mmol/L (38.1 ± 5.0 mmol/L, *n* = 7, Reference 40; also for grey matter 52.1 ± 7.1 mmol/L and white matter 41.8 ± 6.7 mmol/L, *n* = 11,^41^ and grey matter 40.6 ± 2.3 mmol/L and white matter 32.0 ± 3.4 mmol/L, *n* = 34, Reference 42), heart blood, 71 ± 8 mmol/L (79 ± 8 mmol/L, *n* = 10, Reference 43), kidney, 53 ± 27 mmol/L (cortical 58 ± 17 mmol/L and medulla 99 ± 18 mmol/L, *n* = 50,^44^ also for medulla, cortex, and whole kidney 136 ± 7 mmol/L, 72 ± 6 mmol/L, and 93 ± 9 mmol/L, respectively, *n* = 12, Reference 45), and muscle, 31 ± 8 mmol/L (28.4 ± 3.6 mmol/L, *n* = 10, Reference 46, and 26 ± 4 mmol/L, *n* = 10, Reference 47). The literature indicates a range of sodium concentrations with a small standard deviation per study and a more pronounced difference across studies. In a review by Madelin and Regatte, a similar range of values is shown in brain (grey matter 30–70 mmol/L, white matter 20–60 mmol/L), blood (140–150 mmol/L), and muscle (15–30 mmol/L).^8^ The compared TSC values are from reported healthy subjects, as sodium concentration increases in unhealthy tissues, as seen in small vessel brain disease^48^ and cognitively impaired patients.^49^

The multi‐organ TSC values experience a more significant standard deviation than mentioned in the literature. This is most pronounced in the kidney (53 ± 27 mmol/L), and it could arise from a higher standard deviation (Figure 5) combined with the *B*_1_ field corrected TSC outlier (Figure 6), which is not observed in the other organs. Another possibility is the slightly lower sample size (5 versus 7–12) and, more importantly, that we did not correct for water intake before scanning.^50^ Kidney sodium levels are influenced by the water balance, as abstaining from water leads to an increased sodium concentration. This effect has previously been reported in a water loading/water fasting experiment on healthy subjects. Results showed reduced TCS levels in hydrated (1 L water 30 min prior to scan) kidney compared with when abstained from water (no intake for six hours prior to scan).^51^

A more pronounced mean TSC difference was measured in the brain (53 ± 12 versus 38.1 ± 5.0 mmol/L), which could be due to coil placement. We applied a plastic frame with a fixed distance between the two Helmholtz coil elements to avoid the discomfort of having the coils placed directly on the face. Nevertheless, this added distance between the front of the head and the coil. Applying the *B*_1_ correction showed an improvement in signal variation across the brain with a more homogeneous field recovering lost signal in front of the brain. Comparing the results with a multi‐site and multi‐vendor study using a dual tuned (^1^H/^23^Na) head coil shows good agreement in sodium levels (whole brain 53 ± 12 versus grey matter 52.1 ± 7.1 mmol/L and white matter 41.8 ± 6.7 mmol/L).^41^

Although the study reports a lower signal variation, it was statically insignificant in the thigh muscle. TSCs were seemingly unchanged, too, indicating that the effect of *B*_1_ field correction in this organ has little effect. *B*_1_ transmit field effects are reduced in a smaller FOV, and since the diameter of the loop coil elements is 20 cm we expect this to be the explanation.

The Helmholtz coils serve as single loop coils with only one channel element. Nevertheless, the beneficial effects of *B*_1_ field correction are expected to be reported in both single and multi‐channel coils.^52^ Recent developments have shown that multi‐channel acquisitions^53^ alongside parallel imaging or CS^54^, ^55^ could greatly benefit ^23^Na imaging in terms of SNR and speed. ^23^Na multi‐channel coils are often organ‐specific (e.g., the head), and Helmholtz coils’ more flexible application might be beneficial for multi‐organ examinations.

^23^Na imaging was acquired at 3 T; however, using higher field strength would improve SNR significantly.^56^ Systems with a field strength of 7 T are now clinically available, and several publications underline the benefit of clinical applications on these systems.^1^, ^8^, ^10^ Nevertheless, most clinical systems are 1.5 T and 3 T.

There are several limitations to this study. Our study only evaluated the BLOSI method for *B*_1_ correction. A multi‐organ comparison between BLOSI, double angle, and phase‐sensitive techniques would have been a valuable addition to determine clinical feasibility. Lommen et al compared ^23^Na *B*_1_ mapping using phase‐sensitive, BLOSI, double angle, and actual flip angle techniques.^12^ Their work was performed to measure sodium levels in the brain of healthy subjects using a dual tune ^1^H/^23^Na birdcage coil, and the study showed the phase‐sensitive technique to have the best performance. Improved anatomy was based on comparisons with ^1^H images. Such comparisons should never stand alone since ^1^H and ^23^Na images show different contrasts between soft tissues. Nevertheless, the comparison enables a fair estimate of the improvement in the most fundamental anatomical structures, such as the brain, kidneys, cardiac chambers, and muscle circumferences. The acquired low resolution (10 mm, isotropic) ^23^Na images could cause partial volume effects due to the wide point‐spread function. It has been shown that including partial volume effect correction may improve sodium quantification.^57^ However, this was not used in our study. The number of subjects is low and could be increased to enhance statistical power and most probably reduce the standard deviation in the TCS values. Nevertheless, given the scope of evaluating the clinical feasibility of a technical improvement, the number of participants seems reasonable.

## 5 CONCLUSIONS

The study demonstrates the potential of a BLOSI‐based *B*_1_ field correction in multi‐organ ^23^Na images. Organs include the brain, heart, kidney, and thigh muscle. ^23^Na imaging consisted of a quantitative protocol acquired at 3 T within 15 min, including *B*_1_ field imaging. This supports using the fast *B*_1_ field mapping (2 min 7 s) in the clinical setting.

## CONFLICT OF INTEREST

M.V. and R.F.S. are employees of GE Healthcare. The authors report no conflicts of interest. The authors alone are responsible for the content and writing of the paper.
